# Soil microbial functional gene dataset associated with *Agathis australis*

**DOI:** 10.1016/j.dib.2023.109791

**Published:** 2023-11-10

**Authors:** Praveenth Lawrence, Mahajabeen Padamsee, Kevin Lee, Donnabella C. Lacap-Bugler

**Affiliations:** a School of Science, Faculty of Health and Environmental Science, Auckland University of Technology, Auckland 1010, New Zealand; b Manaaki Whenua Landcare Research, 231 Morrin Road, Auckland 1072, New Zealand

**Keywords:** Soil microbiome, *Agathis australis*, Kauri dieback, Geochip 5

## Abstract

*Agathis australis* (New Zealand kauri) is a significant and iconic native tree of Aotearoa New Zealand. Currently, *Phytophthora agathidicida* that causes kauri-dieback disease is killing kauri trees. Only 1% of the New Zealand virgin kauri forest remains [1,2]. Recent studies revealed that many soil-borne microorganisms had been found to systemically boost the defensive capacity of the trees by providing competition to pathogens for nutrient intake, thus preventing pathogen colonization and modulating plant immunity [3,4]. In addition, the root microbiome consists of an entire complex rhizosphere-associated microbes with their genetic elements and interactions that have influenced plant health. To date, very few studies have been conducted to investigate the microorganisms in the kauri soil and possible environmental drivers.

To characterize the functional gene profile in relation to soil microbial diversity of the kauri trees at Auckland Botanic Gardens (ABG), Auckland, New Zealand the GeoChip 5.0 M (Glomics Inc. USA), a microarray-based metagenomics tool, was used. GeoChip 5.0 M comprises of 162,000 probes from 365,000 target genes (coding DNA sequence - CDS), which covers all taxonomic groups (archaea, bacteria, fungi, protists, algae, and viruses) [5]. The ABG has kauri trees that are approximately 20 years old, located in three distinct man-made environments: Native Forest, Kauri Grove, and Rose Garden. We selected two trees from the Native Forest and two from the Kauri Grove for our experiment. Soil samples were collected from the four cardinal points of each tree, at 10 cm depth. Pooled environmental DNA was sent to Glomics (USA) and the data were preprocessed using GeoChip data analysis pipeline described in http://www.ou.edu/ieg/tools/data-analysispipeline.html. Based on the GeoChip data generated from the soil samples, we have detected a total of 946 genes, 4342 taxa, 102 phyla, and 995 genera. The data presented here provide an overview of functional genes associated with kauri soil, which can serve as baseline for other kauri soil microbiome analysis at forest-scale studies. The raw data has been uploaded to Mendeley Data https://doi.org/10.17632/T22NNN385K.1.

Specifications Table

**Table:** 

Subject	Biological Science – Microbiology – Microbiome		
Specific subject area	Soil microbiome, taxonomic and functional diversity, around ∼20-year-old kauri stands in an anthropogenically designed setting.		
Type of data	Table and Figures		
How the data were acquired	Geochip Hybridization 5.0 M platform		
Data format	Raw and analysed		
Description of data collection	Soil samples were collected from four cardinal points under the tree canopy, 100 cm from the kauri tree trunk from four representative trees at Auckland Botanic Gardens, Auckland, New Zealand (Table 1). Environmental DNA was extracted following the CTAB hot-Phenol/Chloroform extraction protocol [6]. GeoChip 5.0 M was used to generate the microarray data. Sample preparation, hybridization, imaging and normalization methods followed Shi et al. 2019.		
Data source location	Institution: Auckland Botanic Gardens City/Town/Region: Auckland Country: New Zealand Latitude and longitude		
	Table 1. Latitude and longitude of the kauri trees sampled.		
	**Sampled Tree**	**Latitude**	**Longitude**
	
	Native Forest Tree 1 (NF1)	−37.00503	174.903210
	Native Forest Tree 2 (NF2)	−37.00487	174.906020
	Kauri Grove Tree 3 (symptomatic Tree) (KG3)	−37.00785	174.906020
	Kauri Grove Tree 5 (Healthy Tree) (KG5)	−37.00781	174.905940
	
Data accessibility	Repository name: Mendeley Data Data identification number: DOI: 10.17632/t22nnn385k.1 Direct URL to data: https://data.mendeley.com/datasets/t22nnn385k/1		

## 1 Value of the Data


 

• This provides baseline data for the taxonomic and functional gene profile of kauri soil from a man-made environment.
  Geochip data can be used by other researchers to compare the core microbial community structure and functional genes among kauri trees from different locations.
• The data may be used as a basis for microbial community structure in comparison with other conifer forest soil. The data can be used as reference data for studies that require soil microbiome baseline data from a controlled environment.

## 2 Data Description

Here we describe a dataset of the number of probes and relative probe intensities associated with different functional gene families and taxa from soil samples which reflects the functional diversity and metabolic potential of microbial community surrounding kauri trees at Auckland Botanic Gardens, Auckland, New Zealand. The dataset consists of four trees labelled NF1 and NF2 for trees from the Native Forest, KG3 and KG5 for trees from the Kauri Grove. The unique probes for each tree sample were annotated to specific gene categories and taxonomic assignments. The lineages and corresponding accession number for each taxonomic assignment were also provided. The microarray Geochip 5.0 M detected a total of 49,005 distinct probe using the current version of GeoChip which relate to 17 different gene categories and 947 gene families. The NF1 and NF2 samples displayed a total of 62,642,872 and 62,344,094 normalized signal intensities of the 85,070 genes probes, respectively. Whilst the KG3 and KG5 samples exhibited a sum of 66,489,421 and 63,035,864 normalized signal intensities from the 89,081 gene probes, respectively. The metal homeostasis gene category had the highest sum of normalised signal intensity out of all the gene categories, with a total of 62,885,341. On the other hand, the category of genes associated with protozoa had the lowest number of detected genes and sum of normalised signal intensity of only 574,856.01 (Table 2).

**Table 2:** Sum of normalized signal intensity detected for each gene category.

	Kauri trees			
Gene Categories	NF1	NF2	KG3	KG5
Antibiotic resistance	12,068,796	12,115,345	12,882,556	12,193,041
Carbon Cycling	8,053,059	7,973,898	8,586,235	8,108,283
Electron transfer	209,475	206,380	223,236	212,018
gyrB	468,646	466,224	499,052	469,816
Metabolic Pathways	395,341	392,802	432,702	410,615
Metal Homeostasis	15,477,246	15,433,706	16,389,624	15,584,765
Microbial Defense	1,804,034	1,773,183	1,914,525	1,818,436
Nitrogen	2,189,887	2,122,839	2,255,765	2,125,689
Organic Contaminant Degradation	4,952,172	4,998,998	5,310,121	5,059,755
Phosphorus	1,167,763	1,157,908	1,226,946	1,172,685
Pigments	1,453,454	1,445,081	1,537,675	1,450,560
Plant Growth Promotion	201,105	207,094	220,547	205,794
Protist	143,697	141,309	146,382	143,468
Stress	10,674,091	10,571,892	11,303,298	10,698,573
Sulfur	1,544,158	1,525,928	1,634,234	1,548,271
Virulence	1,588,532	1,559,628	1,663,101	1,578,306
Virus	251,418	251,879	263,423	255,789

**Total**	**62,642,872**	**62,344,094**	**66,489,421**	**63,035,864**

**Fig 1:**
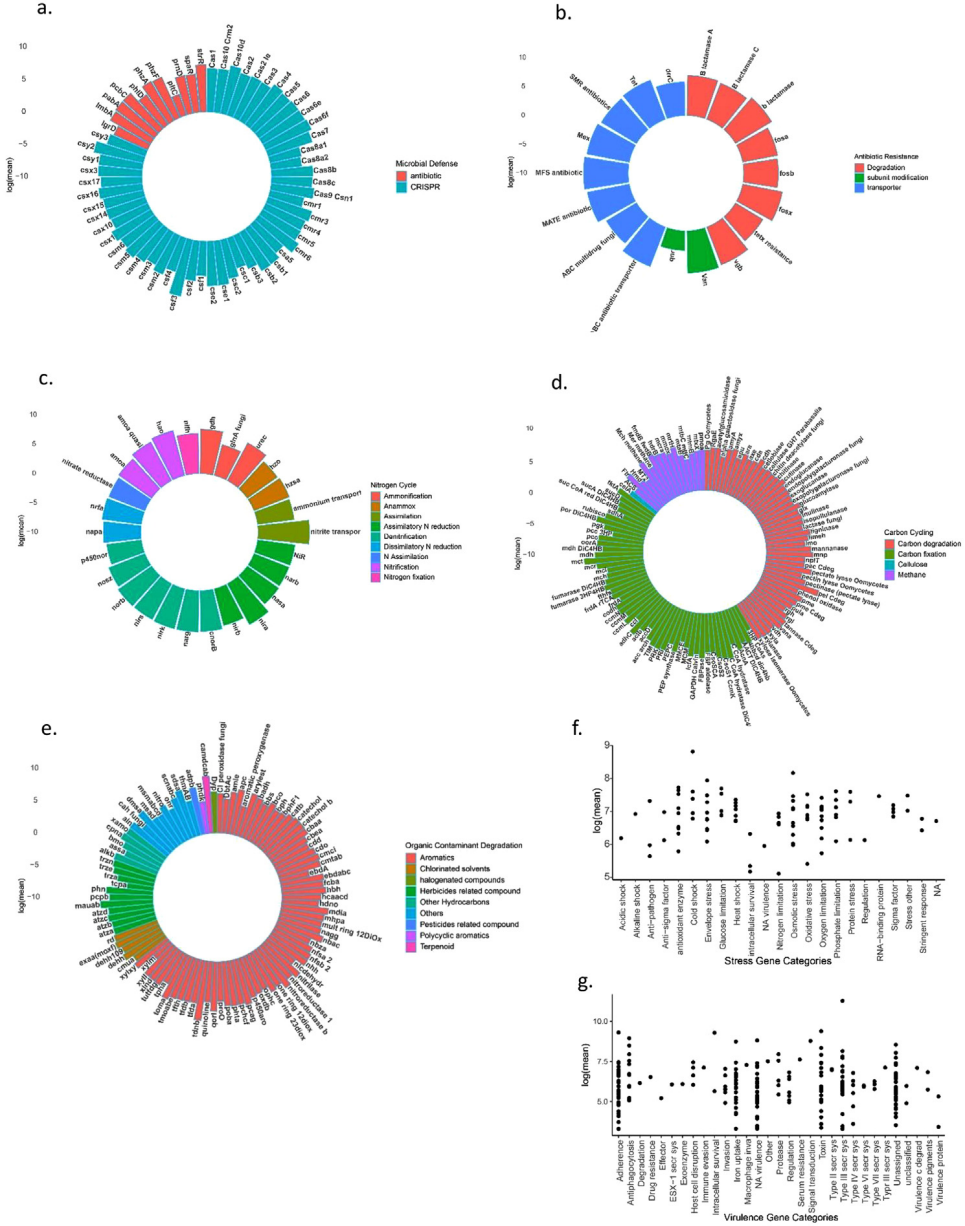
Natural log mean of genes detected in microbial defense gene category (a), antibiotic resistance (b), N cycling (c), organic contaminant degradation (d), carbon cycling (e), stress genes (f) and Virulence genes (g).

In Fig. 1, the natural logarithm (mean) values of genes associated with stress response, nitrogen cycling, antibiotic resistance, microbial defense, carbon cycling, organic contaminant degradation, and virulence gene categories are presented. The dataset show 947 gene families of which 101 stress response genes, 26 nitrogen cycling genes, 18 antibiotic resistance genes, 60 microbial defense genes, 116 carbon cycling genes, and 98 organic contaminant degrading genes were detected from the kauri soil DNA samples (Fig. 1). The microbial defense genes category showed a high detection rate for Cas3, Cas4, and Cas1 genes, while the antibiotic resistance gene category had abundant MFS antibiotic, MEX, and ABC antibiotic transporter genes. The N cycling genes showed that the narg gene for denitrification and the urec gene for ammonification had the highest detection rates. In terms of organic compound degradation genes, the catechol gene from the aromatics gene subcategory and the phn gene from the herbicides related compound gene subcategory had the highest detection rates. The genes amyA and chitinase were found to have the highest of carbon degradation genes. Similarly, the genes ompR and sigma24, which are associated with osmotic stress and sigma factors, respectively, were detected at the highest levels among stress gene categories. Additionally, iro, a gene related to iron uptake, was found to have the highest level of detection among virulence genes.

**Fig 2:**
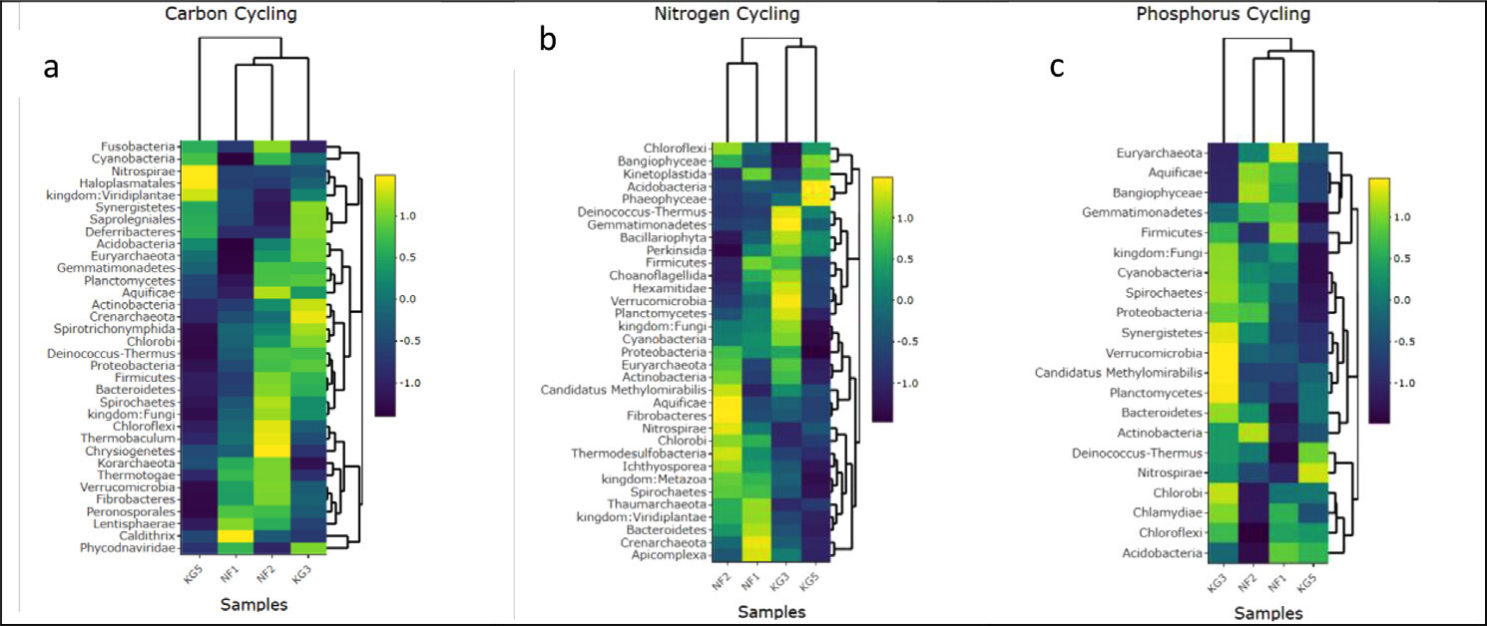
C cycling (a), N cycling (b) and P cycling (c) according to the taxonomy distribution.

**Fig 3:**
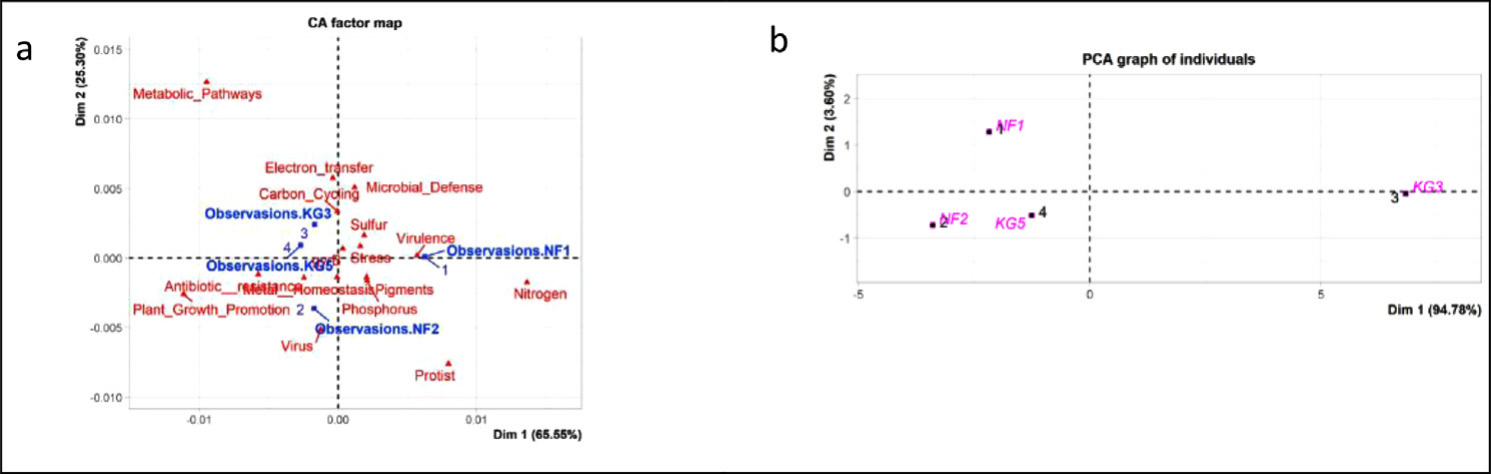
Correspondence analysis and principal component analysis of ABG kauri soil Geochip data.

The dataset also presents the different taxonomic assignment of each probe detected. Fig. 2 shows the taxonomic abundance based on the genes related to C cycling, N cycling, and P cycling. It was identified that the C cycling genes are associated with three archaea phyla, twenty-five bacterial phyla, four fungal classes, and one class from the kingdom Viridiplantae. The detected fungal classes in the samples include Basidiomycota, Ascomycota, Mortierellales, and Mucorales. Additionally, Class Chlorophyta was detected in the kingdom Viridiplantae. The genes related to the N cycle were discovered in various organisms, including 3 phyla of archaea, 18 different bacterial phyla, class Chlorophyta, class Echinodermata, class Ascomycota, and Basidiomycota. Phosphorus cycle genes detected were associated with one archaea phylum, 18 bacterial phyla, 2 orders from class Ascomycota and 3 orders from Basidiomycota (Fig 2). Table 3 displays the alpha-diversities derived from the GeoChip data and Fig. 3 displays both the principal component analysis and correspondence analysis.

**Table 3:** Alpha diversity indices.

	Samples				Mean	Standard Deviation (sd)
	NF1	NF2	KG3	KG5		
Shannon index	4.23	4.15	4.17	4.23	4.20	0.0407
Simpson index	0.98	0.97	0.98	0.98	0.98	0.0027
Species Richness	94.00	99.00	94.00	95.00	95.50	2.3805
	
Observed Species	102					
chao	108.75					
chao.se	7.74

## 3 Experimental Design, Materials and Methods

**Fig 4:**
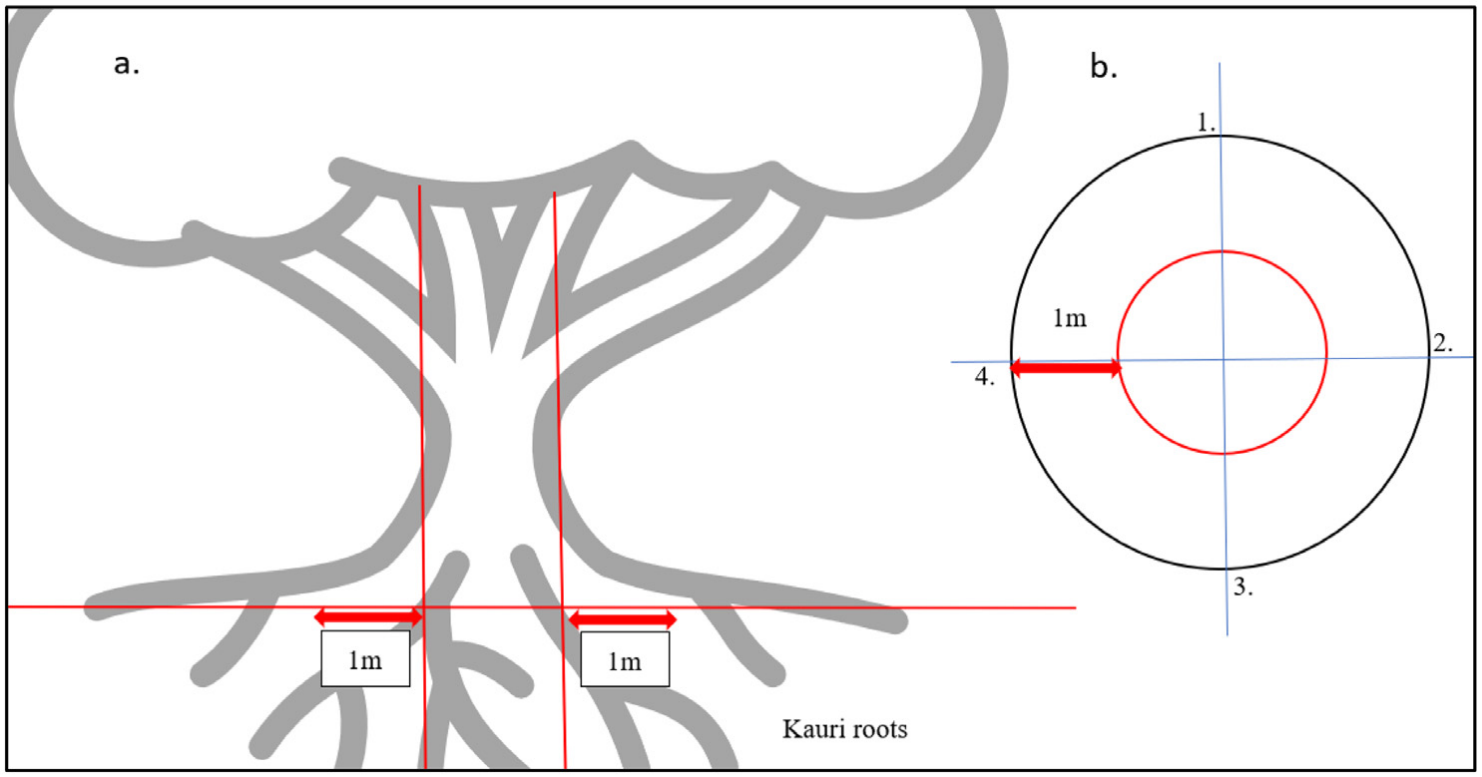
(a) The rootzone of mature kauri tree (b) Sampling points (four cardinal points) from the kauri trunk.

Auckland Botanic Gardens in Aotearoa New Zealand is maintained by the local council and has 23 distinct sections including Native Forest, Kauri Grove, and Rose Garden. The Native Forest section is designed to replicate a natural forest environment and features native trees and plants that are unique to Aotearoa New Zealand, such as kauri tree. The Kauri Grove contains around 100 kauri trees, all of which are about 20 years old. For the experiment, four trees were chosen - two from the Native Forest section and two from the Kauri Grove site. Surface organic matter was removed to expose the soil. Approximately 100 g of soil were collected from the top 10 cm at the four cardinal points, 100 cm from the tree trunk (Fig 4). The roots of kauri trees usually extend three times the distance from the center of the trunk to the edge of the canopy, and they are shallow. Soil samples were stored at −20 °C until processed.

### 3.1 DNA extraction

Genomic DNA was extracted from 0.5 g of soil from each cardinal point following the CTAB hot phenol-chloroform DNA extraction method. In brief, samples were incubated in phosphate buffer, SDS, CTAB, lysozyme and proteinase K in 60 °C water bath for one hour and purified using Phenol-Chloroform. Recovered DNA was quantified using a Quant-iT dsDNA Assay kit (Invitrogen, California USA) on a Qubit 2 Fluorometer (Invitrogen, California USA) according to manufacturer directions. Equimolar of genomic DNA from each cardinal point were pooled and sent to Glomics for GeoChip 5 analysis.

The DNA was purified, by adding 10 µL of NaAc (3 M pH 5.2) (which is 1/10 of the DNA volume), followed by 200 µL of cold 100% ethanol (which is twice the DNA volume). The mixture was thoroughly mixed and then incubated in a negative twenty-degree overnight. The mixture was centrifuged at maximum speed (15,000 g) for 15 min. Supernatant was discarded. 500 µL of 70% cold ethanol was added, then vortexed slowly before being centrifuged at maximum speed (15,000 g) for 15 min. Supernatant was discarded and pellet was air dried for 5 min. The pellet was resuspended in 100 µL nuclease free water.  DNA samples were quantified using a Quant-iT dsDNA Assay kit (Invitrogen, California USA) on a Qubit 2 Fluorometer (Invitrogen, California USA) according to manufacturer directions. Sample purity was assessed using a Nanodrop Spectrophotometer (NanoDrop Technologies Inc., Wilmington, DE). DNA quality was evaluated by the absorbance ratios at A260/280 and A260/230. Only DNA with A260/280 and A260/230 ratios > 1.7 and 1.8, respectively were used for further GeoChip analysis.

### 3.2 Geochip 5.0 experiment

Geochip 5.0 M was manufactured by Agilent (Agilent Technologies Inc., Santa Clara, CA). The fluorescent Cy-3 labeling of DNA was accomplished using the random priming method with Klenow fragment. The DNA was subsequently purified with a QIAquick purification kit from Qiagen, CA, USA according to the manufacturer's instructions and dried. After resuspension of the labeled DNA in DNase/RNase-free distilled water, it was added to the vial containing the lyophilized 10 × aCGH Blocking Agent and hybridization solution containing 10% formamide. The hybridization solution was pipetted into the center of a gasket slide well from Agilent and then covered with an array slide. To ensure better results, SureHyb chamber was closed and the hybridization process was allowed to proceed for 24 h at a temperature of 67 °C in an Agilent Hybridization Oven. Once hybridization was complete, slides were rinsed using Agilent wash buffer at room temperature. Imaging of the array was conducted with the NimbleGen microarray scanner at 633 nm, and data extraction was performed using the Agilent Feature Extraction program, v11.5. The data was extracted and uploaded onto the GeoChip data analysis pipeline (http://www.ou.edu/ieg/tools/data-analysispipeline.html). To ensure accuracy, all arrays in the experiment underwent a two-step normalization and quality filtering process [7,8]. Initially, spots with low-quality were eliminated, which had a signal to noise ratio of less than 2.0. The average signal intensity of the five common oligonucleotide reference standard probes (CORS) was calculated for every subarray. The highest average value among all subarrays was utilized to normalize the signal intensity of samples in each array. For each array, the signal intensity was calculated and the highest value was used to standardize the signal intensity of all spots in that array. This resulted in a normalized value for each spot in every array [9]. The Shannon index and Simpson index were utilized to analyze the diversity of soil microbes and genes, principal component analysis and correspondence analysis was employed to compare the samples. The statistical analyses were conducted in R (version 4.3.0, 2023–04–21) using various packages such as vegan, factoshiny, ggplot2, tidyverse, webr, dplyr, viridis, tidyr, and heatmaply.

## 4 Limitations

It is possible that surface soil may have been mixed in with the samples taken at a depth of 10 cm below the surface. Loss of DNA during extraction processes.

## Ethics Statement

Authors have read and follow the ethical requirements for publication in Data in Brief and confirming that the current work does not involve human subjects, animal experiments, or any data collected from social media platforms.

## CRediT authorship contribution statement

**Praveenth Lawrence:** Data curation, Formal analysis, Investigation, Methodology, Visualization, Writing – original draft. **Mahajabeen Padamsee:** Supervision, Writing – review & editing. **Kevin Lee:** Formal analysis. **Donnabella C. Lacap-Bugler:** Conceptualization, Methodology, Project administration, Supervision, Visualization, Writing – review & editing.

## Data Availability

Soil microbial functional gene dataset associated with Agathis australis (Original data) (Mendeley Data)
